# USING QUALITATIVE ANALYSIS TO DEVELOP HIRING GUIDELINES TO IMPROVE CAREGIVING FOR PEOPLE WITH DEMENTIA AT HOME

**DOI:** 10.1093/geroni/igae098.3925

**Published:** 2024-12-31

**Authors:** Deborah Watman, Jennifer Reckrey

**Affiliations:** Icahn School of Medicine at Mount Sinai, New York, New York, United States; Icahn School of Medicine at Mount Sinai, Icahn School of Medicine at Mount Sinai, New York, United States

## Abstract

Many people living with dementia are being cared for at home by hired paid caregivers. While paid caregivers frequently become an integral part of the care team, little is known about how they are selected by families of people living with dementia. We conducted a secondary analysis of qualitative data collected during two studies that examined family caregiver perspectives on the role of paid caregivers in home-based dementia care (study 1 n=15, study 2 n=9). We developed a codebook focused on emergent themes related to the selection and hiring of paid caregivers, specifically noting practical advice from family caregivers. We analyzed results using thematic analysis. Family caregivers frequently described difficulty setting up care and hiring paid caregivers. This was true for both those who hired paid caregivers privately and those who worked with agencies who provided Medicaid-funded paid care. Many had to cycle through several caregivers, increasing stress for the family, and reducing constancy for the person with dementia. Our findings point to the need for improved hiring guidelines for paid caregivers. Informational materials, including sample interview questions for both paid caregivers as well as family members, can improve the efficiency and quality of the hiring process. It behooves family members, home health agencies, and privately paid caregivers to approach the hiring process deliberately and thoughtfully. Creating and distributing formalized guidelines and protocols can serve as a helpful resource at a stressful time, and improve the overall quality of care for the person with dementia at home.

